# A Mexican Remedy for Dysentery

**Published:** 1893-11

**Authors:** R. T. Knox

**Affiliations:** Gonzales, Texas


For Texas Medical Journal.

### A MEXICAN REMEDY FOR DYSENTERY.

BY DR. R. T. KNOX, M. D., GONZALES, TEXAS.

FOR three years, I was personally a sufferer from chronic dysentery, which is possibly the most irrepressible foe one can have to comtend with. It was at first malarial dysentery, brought about by over work in the sick room, and extreme exposure in a climate filled with poisonous matter, living in it day and night for many days, on one of our most sluggish streams, surrounded by lakes and marshes covered over with quantities of green scum and moss. I was attacked about May 1, 1889, the disease continuing in an uncontrollable manner all summer and fall. The bloody discharges were immense for four months or more, with duodenitis, colonitis and rectitis involving the entire track of the bowels, no medicine controlling, although holding in abeyance the discharges for a time; no diet suitable. Even bread, "the staff of life," was for six weeks utterly ignored. Thousands of prescriptions, or remedies, were proposed, many of which were tried. Loss of flesh and strength were marked, as could be expected.

The best kind of medical attention was given me, both at home and in adjacent cities. While sick at Gonzales, my home, I had the professional care of Drs. J. C. Jones, N. C. Tate, and J. J. Fouts. When at Kerrville, that of Drs. Palmer and Everts, also Dr. Hicks, of San Antonio, my friend Dr. R. H. Harrison, sr., of Columbus, and my son, Dr. T. R. Knox, of Hallettsville, all of whom were untiring in their attention, the last two named leaving their distant homes and business to look after my wants. To these several gentlemen, masters of their calling, I will ever be grateful; they nursed and cared for me like brothers indeed, and here is an offering to their skill and kindnes, all of which was so markedly shown in their treatment of my case.

Every resource was drawn upon by them and my family for my comfort and relief, and everything was done and tried. Irrigation to the bowels with warm water, and at different temperatures; hydrastin lactopeptine, bismuth, opium, salol, petroleum, as also many other medicines by the mouth; nitrate of silver injections, with suppositories of opium, and belladonna, with milk and Mellin's food for diet, change of climate to mountain air for many weeks, thence to the seaside coast for fish diet; yet all afforded but little and temporary relief. Night was the time of greatest disturbance, owing to relaxation and warmth.

Time wore on, and I wore on with it, not being worn out by the disease, nor wearing it out; but from a robust man of 196 pounds I was pulled down to 142 pounds, unfitted for any kind of work or exercise; œdema of the legs very great, impeding the locomotion, causing a "tired feeling" at all times, relieved only and temporarily by frequent bathing with warm water.

Venturing from one article of light diet to another more substantial was risky uncertainty, no fruit or vegetables only the roasted Irish potato, was tried, with the hope of easy digestion. It was well received by the stomach, but proved a scourge when passing along the bowels; so it was abandoned. My friend, Dr. C. E. R. King, of San Antonio, urged me to hope until *peach time*, peaches would cure me. Peach time, however, never came, nor did the cure. This suggestion has much merit, as no doubt the peach juices are good for this condition—the acid it contains. The horse radish root chewed, and the juices only, swallowed, possibly did more good than all else.

Nothing gave more than temporary relief; discharge after discharge of quanties of bloody matter, accompanied with much pain. I was hungry all the while, yea, ravenously so, and this in a land of plenty, and to spare.

And now, if you will pardon my long statement, comes the strangest part of this history, and however it may be to you, the part most enjoyable to my patient. An intelligent farmer, Wm. Mahon, a friend from the country who came to see me, who claimed he had been a long sufferer in a like manner, and had been cured, wanted me to try the remedy he used. This same thing had been suggested to me by Mr. J. R. Roschell, druggist of this place. He had with him the medicine in the form of a bush, or shrub, a native product or growth here. From this was made a tea, or infusion, and this was drunk freely before each meal eaten; he insisted on my trying its merits. He being extravagant in its praise, obedience was reluctantly given to his wishes. He brought me a quantity of the shrub, from which a quantity of tea was made, having the appearance of beautiful sparkling beer. Of this I drank free quantities before each meal. But oh! for Marah's bitter waters to put a sweet taste in my mouth, as one would fancy it was no way comparable to this bitter dose; bitter was no name for it, it was *immensely* bitter.

I met several intellelligent Mexicans, and endeavored to learn the name of this growth, which in the Mexican language is *Bisbiranda Armagosa*, while one told me it was known by the Americans as "chapparo bush," while each said for diseased bowels it was "bueno" (good), all insisting on this as a fact.

Its use was continued earnestly and persistently. After three days the condition of the bowels was found to be much improved.

The medicine was kept up, and after two weeks' use of this simple remedy the bowels were under control, digestion quite good, the actions consistent and partially moulded, a condition which did not exist only a very few times during the three years. Sleep was good, undisturbed through the entire night; strength followed. There was an appreciable alterative action going on, as the drug seems to possess this beneficial property.

[The above is extracted from a paper prepared by Dr. Knox for the meeting of the Texas State Medical Association held in Waco April, 1892. At that time he was still suffering, but under the use of this medicine was improving rapidly, and hoped at next meeting, he said, to exhibit his patient (himself) sound and well. The doctor fully recovered his health and strength, and attributes to cure to the use of the *Chapparo Amaroso.* It is a thorny shrub, indigenous to Texas and Mexico. Recently a remarkable cure of chronic dysentery has been effected in Austin by the use of this remedy, sent to the father of the patient, Capt. Oliphant, by Dr. Knox. A further trial of the drug is being made by several physicians, and the JOURNAL hopes to be able to give the results at some future time.—ED.]

